# Reactive species generated by a cold atmospheric plasma inactivate AAV through oxidative and nitrosative stress

**DOI:** 10.1038/s41598-026-52499-3

**Published:** 2026-05-09

**Authors:** Aleksandra Y. Lavrikova, Aline Aebi, Fabio Avino, Elodie Y. Dubois, Bernard L. Schneider, Ivo Furno

**Affiliations:** 1 Ecole Polytechnique Fédérale de Lausanne (EPFL), Swiss Plasma Center (SPC), Lausanne, 1015 Switzerland; 2 Ecole Polytechnique Fédérale de Lausanne (EPFL), Bertarelli Platform for Gene Therapy, Ch. des Mines 9, Geneva, 1202 Switzerland

**Keywords:** Cold atmospheric plasma, Dielectric barrier discharge, Reactive nitrogen species, AAV, Virus inactivation, Biological techniques, Biotechnology, Microbiology

## Abstract

Cold atmospheric plasmas offer a non-thermal chemical-free technology of bio-decontamination. Compared to their antibacterial effects, the plasma-based antiviral mechanisms remain poorly studied, hampering their promising widespread application. Here, the robust Adeno-Associated Viral (AAV) vector was used as a model to investigate the plasma virucidal effects. AAV inactivation was analyzed by *in vitro* testing of virus infectivity, vector genome quantification, and vector capsid integrity. It was found that plasma treatment for 10 min resulted in at least a 3-log_10_ reduction in AAV6 infectious activity. The acidification and enrichment in nitrites and nitrates characterized plasma-activated phosphate-buffered saline. The results suggest that oxidative and nitrosative damage to the viral capsid proteins, along with partial genome degradation, accounted for the loss of AAV6 infectivity. Collectively, these findings demonstrate the inactivation capacity of plasma-generated reactive nitrogen species for AAV6 and support their potential use in disinfection strategies applicable to non-enveloped viruses such as AAV.

**Supplementary Information:**

The online version contains supplementary material available at 10.1038/s41598-026-52499-3.

## Introduction

Cold atmospheric plasmas (in the text referred to as ‘plasmas’) are a promising approach to cope with viruses^1–3^. Plasma is a partially ionized quasi-neutral gas comprising ions, electrons, UV photons, electromagnetic field, and reactive species such as radicals, excited and ground-state molecules^4^. Plasma generates a high concentration of biocidal reactive oxygen species (ROS) and reactive nitrogen species (RNS), including ozone O_3_, singlet oxygen ^1^O_2_, superoxide radical O_2_^•−^, nitric oxide NO, nitrogen dioxide NO_2_, nitric acid HNO_3_, nitrous acid HNO_2_, peroxynitrite ONOO^−^, hydrogen peroxide H_2_O_2_ and hydroxyl radical •OH, while maintaining a low temperature of both ROS/RNS and neutral particles^5^. It reflects a great deal of recent attention to plasma over traditional methods of thermal and chemical bio-decontamination for its use in biomedical, agricultural, and food-processing industries^6,7^. Yet, studies on plasma-virus interactions are scarce compared to those related to bacteria inactivation, leaving many open issues regarding the mechanisms of plasma action on viruses. From a biological aspect, viruses exhibit far greater diversity than bacteria, especially in terms of genetic and functional variety. From a physical aspect, the diversity of plasma sources, including plasma jets, gliding arc discharges, streamer discharges, and dielectric barrier discharges (DBDs), together with different operating conditions, makes the comparison of plasma-generated mixtures of reactive species problematic. Together, this poses a huge complexity in establishing the plasma-generated reactive species responsible for virus inactivation.

Most studies indicate the damages to virus coat proteins or nucleic acids to be the main cause of virus inactivation by plasmas. For example, inactivation of λ bacteriophage (non-enveloped double-stranded (ds) DNA virus) by DBD was largely caused by damages to surface coat proteins, while DNA damage barely contributed to inactivation^8^. Similarly, DBD-treated *ϕ*X174 bacteriophage (non-enveloped single-stranded (ss) DNA virus) primarily underwent coat protein destruction followed by DNA damage^9^. A more recent study showed that DBD treatment of *ϕ*X174 bacteriophage, T4 (non-enveloped, dsDNA virus), and MS2 (non-enveloped ssRNA virus) induced damages to both proteins and nucleic acids. ^1^O_2_ and O_2_^•−^ played the major role in inactivation^10^. Similarly, ^1^O_2_ and ONOO⁻ were found to be the main reactive species for feline calicivirus (FCV, non-enveloped ssRNA virus) inactivation by a DBD plasma torch^11^. The importance of ROS was shown for efficient inactivation of Tulane virus (non-enveloped ssRNA virus). DBD treatment led to virus reduction on the lettuce by 1.3 log PFU g^− 1^; however, treatment in a lowered O_2_ atmosphere decreased the plasma efficacy^12^. Another study revealed that RNS, most likely acidified nitrites, are responsible for the inactivation of FCV suspended in deionized water or NTE-buffered solution by DBD treatment. The inactivation of FCV suspensions positively correlated with a drop in pH, resulting in higher inactivation in deionized water (4.3 log, pH 2.7) than in NTE buffer (0.9-2 log, pH 5.5)^13^. In contrast, HCoV-229E (enveloped ssRNA virus) underwent 4 log reduction in DMEM at pH 7 upon µ-DBD treatment, which generated NO_x_ and H_2_O_2_^14^. Combined effects of a strong electric field, radical etching physical damage, and ROS/RNS interaction with proteins were suggested to inactivate 3.03 log of vesicular stomatitis virus (enveloped ssRNA virus) and 99.3% of SARS-CoV-2 pseudovirus when treated by the flexible Surface Dielectric Barrier Discharge (SDBD)^15^. Zimmermann et al. used surface micro-discharge to inactivate adenovirus (non-enveloped dsDNA virus). In a volume of 20 µl of phosphate-buffered saline (PBS), adenovirus underwent a 6 log inactivation and inhibition in replication after 240 s treatment. Destruction of the capsid was presumed to be the main mechanism of inactivation rather than destruction of DNA. Interestingly, virus inactivation was greater at higher virus concentration, probably due to the increased likelihood of virus encountering dissolved reactive species^16^. The opposite effect was found by Ahlfeld et al. during treatment of Norovirus (non-enveloped ssRNA virus) by surface micro-discharge. Lower initial virus quantities and higher dilutions of the stool suspensions led to increased virus inactivation^17^. The recent insightful review by Zver et al. on viruses’ inactivation by plasmas in water solutions revealed no correlation between D-value and initial virus concentration and between D-value and plasma power in a range of 6–400 W·ml^− 1^^18^.

To further investigate the potential of plasma-based virus inactivation, the current work studied the effects of the SDBD treatment on virus suspension in PBS. Due to their high physical stability, Adeno-Associated Viral (AAV) vectors were used as a biological model to assess the virucidal efficacy of plasma. Discovered in 1965 as a contaminant in adenovirus preparations^19^, AAV is a non-enveloped virus containing an ssDNA genome with inverted terminal repeats (ITRs) at both ends. AAV has emerged as one of the dominant gene transfer vectors nowadays. There are seven approved AAV gene therapies including Glybera (2012), Luxturna (2017), Zolgensma (2019), Hemgenix (2022), Upstaza (2022), Elevidys (2023), and Roctavian (2023)^20^. The rising demand for AAV production raises concerns about potential vector dissemination due to insufficient decontamination, despite a safe nature of the vector system. As chemical oxidant-based disinfectants, sodium hypochlorite and potassium peroxymonosulfate show efficient capsid and genome denaturation of AAV. Peracetic acid is effective against AAV1 but not AAV5. While 70% ethanol and 1.5% hydrogen peroxide are not effective in destroying AAV^21^. Regarding physical decontaminants, UV radiation, gamma radiation, photodynamic therapy, X-rays, and plasmas have shown their efficacy against number of viruses^22^. Among others, UV exposure for 10 min causes a 10,000-fold reduction in AAV activity^23^, and 1 h autoclave treatment at 121 °C inactivates AAV^24^. To the best of the author’s knowledge, no published study is available on the efficacy of plasma against AAV to date.

In this work, the recombinant AAV6 vector was directly treated by SDBD and virus inactivation was evaluated based on measurements of vector infectivity, DNA degradation, and capsid destruction. The analysis of the physicochemical properties of Plasma-Activated PBS (PAPBS) underscores the potential of RNS to achieve at least a 3-log_10_ reduction in AAV6 activity, as shown for the first time. The findings of this study provide significant insights into plasma-virus interactions and illustrate the plasma’s potential in AAV disinfection practices.

## Results

### Virus infectivity

**Fig. 1:**
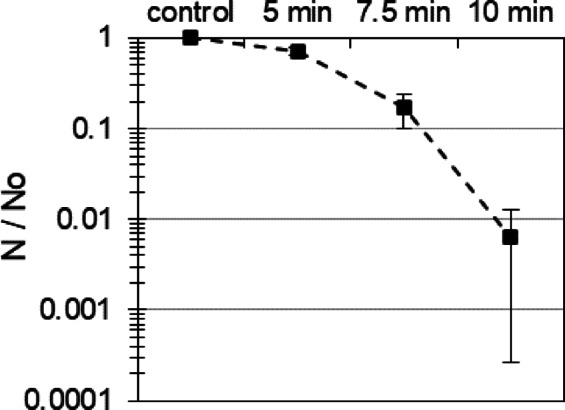
The time-dependent AAV6 inactivation upon plasma treatment. *N*, the amount of transduction units (TUs) after treatment; *No*, the amount of TUs in an untreated control group. *P* < 0.0001. Increased error bar at 10 min treatment time reflects the high sensitivity of the *N / N*_*0*_ ratio to low-frequency events as *N* approaches zero.

Plasma treatment for 5, 7.5, and 10 min caused a time-dependent loss of AAV6 infectivity tested on HEK293 cells, causing 1.4-, 6.7-, and 811.5-fold decrease, respectively (Fig. 1). Interestingly, the infectivity of the virus further decreased when plasma treatment was followed by freezing at − 80 °C. The combined effect of plasma treatment and freezing resulted in an infectious virus load decrease by a factor of 37.5, 280.0, and 3763.2 after 5, 7.5, and 10 min treatment, respectively (data not shown). This phenomenon can help to study the plasma-virus interactions and must be further explored.

### Virus particle integrity

**Fig. 2:**
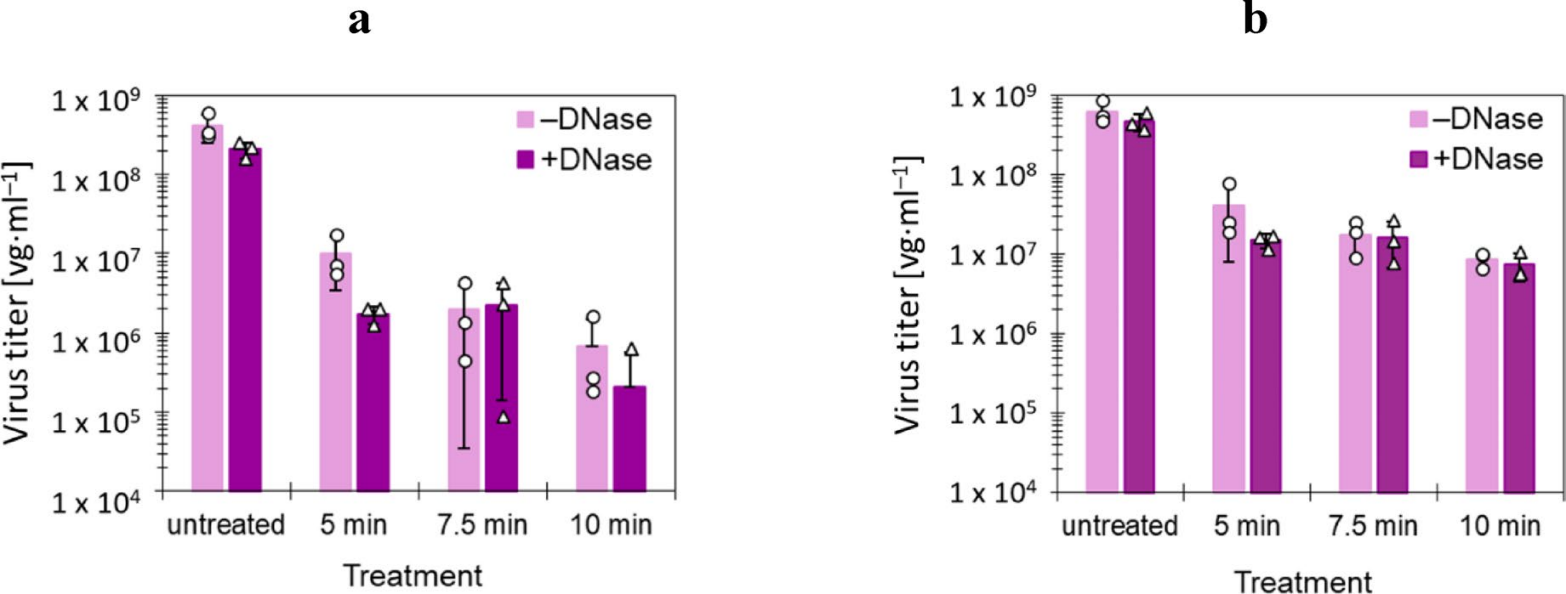
The DNA degradation of plasma-treated AAV6 quantified by dPCR on (**a**) β-globin and (**b**) inverted terminal repeat (ITR) sequences of DNA without (–DNase) and with (+ DNase) the pre-treatment step by DNase I. Values are presented as the mean ± standard deviation (error bars). Circles (○) and triangles (△) represent biological replicates. No statistical difference (*P* > 0.05) was found between –DNase and +DNase conditions among all samples. The statistical significance between untreated controls and all treated samples was found (*P* ≤ 0.05).

The digital Polymerase Chain Reaction (dPCR) measurement was used to determine the concentration of genome-containing AAV6 particles. A decrease in virus titer, expressed as vector genome per ml (vg**·**ml^− 1^), reflects a degradation of encapsidated viral DNA (vDNA) after plasma treatment (Fig. 2). To further assess genome concentration as a function of capsid integrity, viral titers were determined with (+ DNase) and without DNase I treatment (–DNase) before DNA extraction. DNase I was expected to degrade the released vDNA in case of particle integrity breakdown upon plasma treatment. Although plasma caused a slight loss of viral particle integrity, no statistical difference (*P* > 0.05) was found between –DNase and +DNase conditions, indicating that most of vDNA detected by dPCR was contained within AAV6 particles. The AAV6 vector titer measured using the single-stranded genomic β-globin sequence decreased from 2×10^8^ vg**·**ml^− 1^ to 2×10^5^ vg**·**ml^− 1^ (1000-fold) upon 10 min of plasma treatment (Fig. 2a). Viral DNA integrity of the double-stranded ITR sequence was less affected, as 10 min of plasma treatment caused a decrease from 4.5×10^8^ vg**·**ml^− 1^ to 7.2×10^6^ vg**·**ml^− 1^ (62.5-fold decrease) (Fig. 2b).

### Virus capsid degradation

To further assess the effects of plasma treatment on vector integrity, the AAV6 vector suspension was analyzed by TEM to visualize capsid structure. As the AAV6 vector purification and concentration process did not include any step to enrich the suspension in genome-containing particles, the proportion of full particles was low (< 20%) in all conditions and was not quantified, considering the small vector sampling. In this respect, the novel quantitative electron microscopy (QuTEM) approach for distinguishing full, partial, and empty AAV capsids based on their internal density is promising for related studies^25^.

**Fig. 3:**
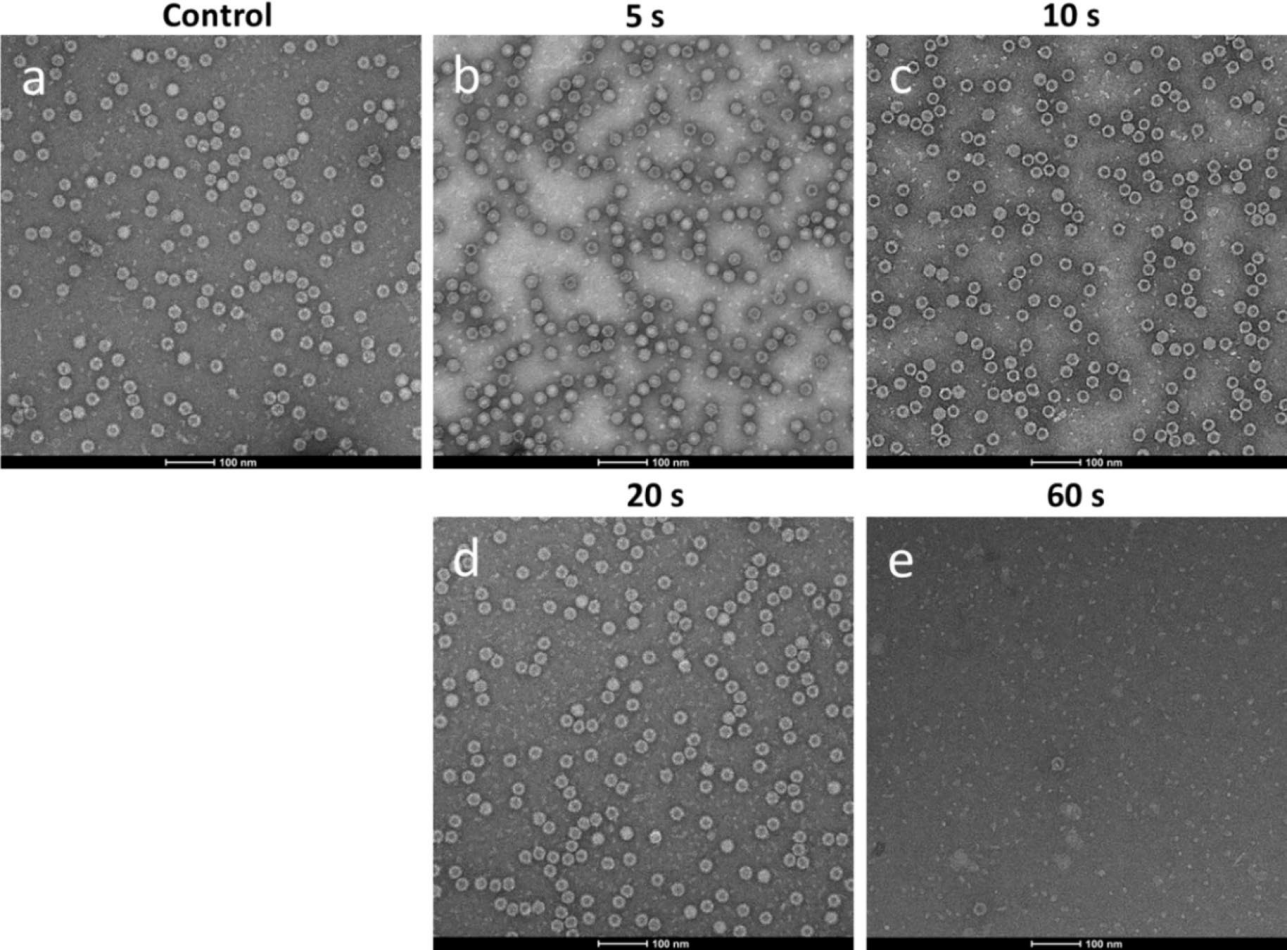
Negative stain TEM images of AAV6 particles. (**a**–**d**) Full (bright spheres) and empty (spheres with a darker spot) viral particles. (**e**) After 60 s of treatment, the suspension contained mainly debris of the collapsed viral particles.

Figure 3b–d shows that there were no major damaging effects on AAV6 capsids after plasma treatment for 5, 10, and 20 s. Both full and empty capsids retained their integrity. However, a longer 60 s treatment induced collapse of AAV6 particles, and only capsid debris most likely corresponding to denatured viral proteins were observed by TEM in this condition (Fig. 3e). Importantly, the direct correlation of the TEM with the dPCR or infectivity results is not possible due to different treatment parameters. Treatment of a 50 µl sample for 60 s here would approximately correspond to a 20 min treatment of the original 1 ml sample used to estimate virus infectivity (Fig. 1) and DNA degradation (Fig. 2). Nonetheless, the TEM findings suggest the total virus degradation in case of extended plasma treatment times.

### Physicochemical properties of PAPBS

**Fig. 4:**
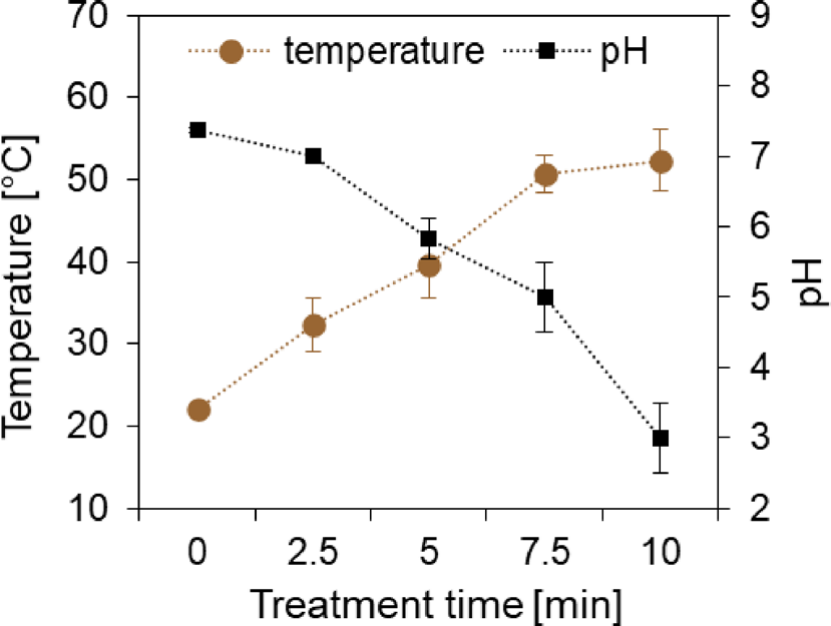
Temperature and pH of PAPBS as a function of plasma treatment time.

The pH of PAPBS gradually decreased from an initial 7.4 value to 3 after 10 min of plasma treatment (Fig. 4). The drop in pH in liquids exposed to air plasmas is related to gas-phase reactions, followed by the dissolution of gaseous NO_x_, and the formation of nitrous acid HNO_2_ and nitric acid HNO_3_^26^. The temperature of PAPBS gradually increased and reached 52 °C by 10 min treatment (Fig. 4).

**Fig. 5:**
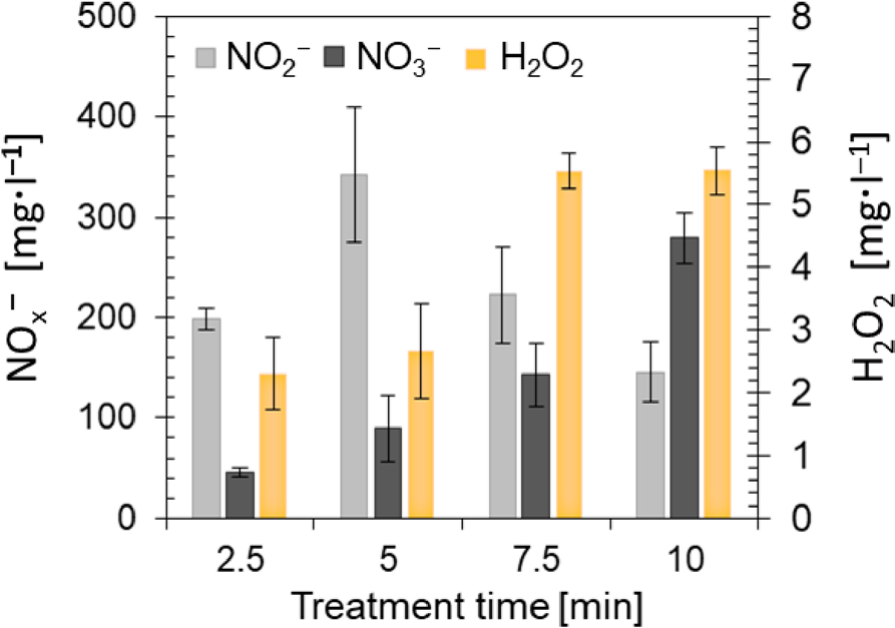
Concentrations of NO_2_^−^, NO_3_^−^, and H_2_O_2_ accumulated in PAPBS as a function of plasma treatment time.

The SDBD operated at a voltage of 8.8 kV generated predominantly RNS with negligible amounts of H_2_O_2_ in treated deionized water^27^ and culture media^28^. Similarly, in the current study, PAPBS accumulated mostly long-lived RNS, namely nitrites NO_2_^−^ and nitrates NO_3_^−^, during 10 min of plasma treatment (Fig. 5). The concentration of H_2_O_2_ reached a maximum of 5.5 mg·l^− 1^ after 10 min (Fig. 5). NO_2_^−^ and NO_3_^−^ are formed from the solvation of the gas-phase nitrogen dioxide NO_2_ (Eq. 1) or via oxidation by nitric oxide NO (Eq. 2)^29^. This induces the drop in pH, which in turn, leads to the disproportionation of NO_2_^−^ into NO_3_^−^ (Eq. 3)^30^. Thus, an initial increase of NO_2_^−^ concentration to 342 mg·l^− 1^ in 5 min treatment was observed, followed by its gradual decrease to 222 mg·l^− 1^ and 145 mg·l^− 1^ in 7.5 min and 10 min, respectively (Fig. 5). Accordingly, the NO_3_^−^ concentration increased during treatment from 46 mg·l^− 1^ in 2.5 min to 279 mg·l^− 1^ in 10 min treatment (Fig. 5).

1 $${\mathrm{NO}}_{{{\text{2 }}({\mathrm{aq}})}} + {\text{ NO}}_{{{\text{2 }}({\mathrm{aq}})}} + {\text{ H}}_{{\mathrm{2}}} {\text{O }} \to {\mathrm{NO}}_{{\mathrm{2}}} ^{ - } + {\text{ NO}}_{{\mathrm{3}}} ^{ - } + {\text{ 2H}}^{ + }$$

2 $${\mathrm{NO}}_{{({\mathrm{aq}})}} + {\text{ NO}}_{{{\text{2 }}({\mathrm{aq}})}} + {\text{ H}}_{{\mathrm{2}}} {\text{O }} \to {\text{ 2NO}}_{{\mathrm{2}}} ^{ - } + {\text{ 2H}}^{ + }$$

3 $${\mathrm{3NO}}_{{{\text{2 }}({\mathrm{aq}})}} ^{ - } + {\text{ 3H}}^{ + } \to {\text{ 2NO}}_{{({\mathrm{aq}})}} + {\text{ NO}}_{{{\text{3 }}({\mathrm{aq}})}} ^{ - } + {\text{ H}}_{{\mathrm{3}}} {\mathrm{O}}^{ + }$$

4 $${\mathrm{HNO}}_{{\mathrm{2}}} \rightleftharpoons {\mathrm{NO}}_{{\mathrm{2}}} ^{ - } + {\text{ H}}^{{ + ~}} \,\,\,\,\,\,\,\,\,\,\,\,\,{\mathrm{pK}}_{{\mathrm{a}}} = {\text{ 3}}.{\mathrm{29}}$$

At pH values below 5.5, nitrous acid HNO_2_ is formed from nitrites. Therefore after 7.5 min of plasma treatment, NO_2_^−^ existed in acidified form and NO_2_^−^ ions were involved in an equilibrium reaction with HNO_2_ (Eq. 4).

## Discussion

AAV is one of the most widely used vectors in gene therapy due to its robustness. The icosahedral capsid, made of tightly packed viral proteins, effectively protects the ssDNA genome of AAV against solvents like ethanol. Unlike enveloped viruses (e.g., influenza, HIV, or coronaviruses) that have a lipid membrane, which is readily disrupted by ethanol by dissolving the lipids and causing the virus to disassemble, non-enveloped AAV remains unaffected. Strong oxidizing agents, such as bleach or potassium peroxymonosulfate, as well as physical treatments such as UV and autoclaving, can be used to inactivate AAV. This study explored the plasma-based virus inactivation by direct treatment of the robust recombinant AAV6 vector resuspended in PBS by SDBD.

In our investigation, the plasma-treated AAV6 exhibited a substantial loss of infectivity, with at least a 3-log reduction in infectious dose, corresponding to the limit of detection (Fig. 1). Current scientific opinion deems antiviral processes as efficient if reaching at least ≥ 3-log_10_ reduction of infectious viruses within ≤ 10 min contact time with disinfectants^31^; meanwhile, the European standard EN 14,476, revised in 2025 to EN 14476:2025 maintains a stricter threshold of ≥ 4-log_10_ reduction for virucidal claims in medical and hygiene settings^32^. Interestingly, the virus pre-exposed to plasma lost its stability at standard − 80 °C storage conditions. To better understand the plasma virus inactivation pathway, the integrity of two major components of the virus, the capsid and the genome, were examined. The morphology of the plasma-treated AAV6 suggests the viral capsid remained assembled upon short treatment durations that represented our samples (Fig. 3). Extended treatment time showed the possibility of virus particles collapse. The number of intact vDNA was reduced by ~ 2–3 log as compared to the initial genome titer (Fig. 2). Therefore, the results suggest that AAV6 exposed to plasma experienced a gradual time-dependent decrease of infectivity predominantly via damages to the capsid without loss of its integrity, and partially via internal genome disintegration.

AAV is a highly stable vector, retaining infectivity over a range of pH values and temperatures^23,33^. However, pH-related structural changes have been reported for AAV1 and AAV6 in which the α-helical structure of capsid proteins was gradually lost when pH decreased from 7.5 to 4^34^. Nevertheless, we believe that the drop in pH alone is unlikely to be the main factor affecting AAV6 integrity and infectivity in our experiments, as the capsid was transiently exposed to acidic pH 3 conditions during the elution step of AAV6 chromatography purification, confirming its stability at low pH^35^. The melting temperature (*T*_*m*_) of AAV capsids lays in the range from 66.5 °C to 89.5 °C. The *T*_*m*_ of the full and empty AAV6 capsids in PBS is 77.5 and 78.5 °C, respectively^36^. Moreover, Kostelic et al. revealed a dependence of AAV thermal stability on the buffer conditions. The AAV8 capsids dissociated at 71 °C in PBS at pH 7, while 65–70 °C was sufficient to dissociate the capsids at pH 5, and 40–45 °C at pH 3^37^. Interestingly, the same study reports that AAV2-CMV-GFP and AAV8-CMV-GFP particles first ejected their genome at 55 °C followed by capsid disassembly at 70–75 °C. Lins-Austin et al. reported acidification to pH 5.5 increased thermal stability of AAV1, AAV2, and AAV8; while pH 4 decreased thermal stability of these AAV capsids. On the contrary, AAV5 decreased its thermal stability after acidification^38^. Furthermore, Ye et al. identified specific AAV8 capsid regions that responded to acidic pH and temperature, triggering DNA release^39^. Thomas et al. found that 55 °C induced a reduction in AAV6.2FF infectivity by 30 min with a continual decline over time^40^. Based on these findings, it is therefore unlikely that the observed virucidal effects in our experiments were associated solely with the temperature increase of PAPBS to 52 °C. Similarly, Aboubakr et al. proposed that changes in pH and temperature as well as the formation of H_2_O_2_ were not responsible for the FCV inactivation by the plasma jet^41^. While synergistic effect between low pH and high temperatures on AAV6 stability remains unexplored, based on abovementioned examples with other AAV serotypes, such effect cannot be excluded in our study.

The capsid plays a crucial role in the initiation of a successful infection. AAV consists of an icosahedral protein capsid composed of 60 copies of three viral proteins (VP1, VP2, VP3). Following initial binding to cell surface glycans (e.g., heparin sulfate, N-linked sialic acids, galactose, etc.), the entry of AAV into target cells is mediated by interactions with co-receptors, such as AAVR or the epidermal growth factor receptor in the case of AAV6^42,43^. A conformational change of the protein coat subunits is likely to render the virus non-infectious since both attachment to the host cell and viral entry into the host cell are functions of the capsid. For instance, Velebit et al.. speculated that O_3_ and NO_2_ induced denaturation of capsid proteins of murine norovirus and hepatitis A virus (both are non-enveloped ssRNA viruses) followed by their penetration into viral particles and eventual virus inactivation^44^. Patel et al. demonstrated a high antiviral capacity of NO_x_-PAW (Plasma-Activated Water) against vaccinia virus (enveloped dsDNA virus), revealing the interaction pathway between RNS and viral surface proteins. Namely, DBD-generated NO, NO_2_^−^, NO_3_^−^, and N_2_O induced modifications of the surface viral entry proteins A27 and H3, leading to the abrogation of virus internalization via micropinocytosis in host cells^45^. Similarly, Khanikar et al. showed that ROS triggered the deactivation of the spike protein of the SARS-CoV-2 virus (enveloped ssRNA), inhibiting its binding to the human ACE2 protein^46^.

In our experiments, the SDBD generated N_2_O, NO_2_, and O_3_ in the gas phase^27^ that led to the formation of predominantly NO_2_^−^ and NO_3_^−^ in PAPBS (Fig. 5). Even though the plasma generated a relatively low H_2_O_2_ concentration in PAPBS, its reaction with NO_2_^−^ might have led to the formation of peroxynitrite ONOO^−^. Under acidic conditions, plasma-generated NO_2_^−^ and H_2_O_2_ form peroxynitrous acid ONOOH (Eq. 5), which is in equilibrium reaction with peroxynitrite anion ONOO^–^ (Eq. 6)^47,48^. In this way we assume that in PAPBS generated for 10 min with pH 3, the reaction (Eq. 5) occurred. At pH 3, a significant portion of NO_2_^−^ exists as HNO_2_, which is the required reactive form, creating chemically favorable conditions for ONOOH formation, even with low H_2_O_2_ concentration. Taken 5.5 mg·l^− 1^ (0.16 mM) of H_2_O_2_ and 145 mg·l^− 1^ (3.15 mM) of NO_2_^−^ in our 10 min sample, gives a ~ 20-fold molar excess of NO_2_^−^ making H_2_O_2_ limiting reagent that must have been completely consumed (Eq. 5). In turn, unstable ONOOH might have decayed into powerful oxidants hydroxyl radical (•OH) and nitrogen dioxide radical (•NO_2_) (Eq. 7) or turned into NO_3_^−^ (Eq. 8)^49^.

5 $${\mathrm{NO}}_{{\mathrm{2}}} ^{ - } + {\text{ H}}_{{\mathrm{2}}} {\mathrm{O}}_{{\mathrm{2}}} + {\text{ H}}^{ + } \to {\text{ ONOOH }} + {\text{ H}}_{{\mathrm{2}}} {\mathrm{O}}$$

6 $${\mathrm{ONOOH}} \rightleftharpoons {\mathrm{ONOO}}^{{-}} + {\text{ H}}^{{ + ~}} \,\,\,\,\,\,\,{\mathrm{pK}}_{{\mathrm{a}}} = {\text{ 6}}.{\mathrm{8}}$$

7 $${\text{ONOOH }} \to {\text{ }} \bullet {\text{OH }} + {\text{ }} \bullet {\mathrm{NO}}_{{\mathrm{2}}}$$

8 $${\text{ONOOH }} \to {\text{ H}}^{ + } + {\text{ NO}}_{{\mathrm{3}}} ^{ - }$$

PAPBS resulted in the formation of nitrites that exist in the form of nitrous acid in an acidic pH. We suggest plasma-generated HNO_2_ as an evident RNS capable of damaging AAV6 since it acts as an oxidant and induces nitrosylation, nitrosation, and oxidation of proteins, fatty acids, and vDNA. Viral capsids are permeable to small molecules like HNO_2_, that can therefore reach the nucleic acid content without capsid disruption^50^. Nitrous acid is a well-known mutagen that leads to deamination of nucleobases in RNA and DNA: adenine to hypoxanthine, cytosine to uracil, and guanine to xanthine^51^. Deamination of nucleobases by HNO_2_ was shown to induce inactivation of the RNA of tobacco mosaic virus and of the bacteriophages T2, T4, and *ϕ*X174^52^. Moreover, HNO_2_ caused mutations in all four nucleobases of bacteriophages S13 and *ϕ*X174, which are ssDNA viruses like AAV^53^. Lytle and Ginoza reported the inactivation of *ϕ*X174 by sublethal alterations of both vDNA and coat proteins caused by HNO_2_ action^54^. Based on genome quantification, we hypothesize that HNO_2_ played a significant role in vDNA deamination in our experiments. However, the partial reduction in the genome titer (by ~ 2–3 log) measured by dPCR suggests that genomic deterioration was not the sole factor in AAV6 inactivation.

Thus, the oxidizing capacity of all RNS present in our system suggests that RNS-induced oxidative stress of VPs is the major mechanism of AAV6 inactivation by plasma. Plasma-generated RNS can penetrate viral structures^10,55^, leading to oxidation of amino acids in capsid proteins^56^, oxidative cleavage of protein backbones^57^, and overall structural damage or degradation of VPs^45,46,58^. Besides their oxidative properties, NO_2_ and ONOO^−^ induce nitration of proteins and DNA^49,59^. ONOO^−^ causes protein tyrosine nitration, protein oxidation, DNA oxidation and nitration^60^. ONOO^−^ has previously been shown to exhibit antiviral activity against several viruses^61^.

In conclusion, this study demonstrates the AAV6 inactivation resulted from primarily RNS-induced oxidative and nitrosative stress to the viral capsid proteins, accompanied by the internal viral genome damage, related but likely not limited to the deamination of vDNA by HNO_2_. The findings propose a new strategy for AAV6 disinfection through plasma-generated RNS, which might be applied by either direct plasma treatment or potentially by indirect treatment with PAW. In summary, the outcomes of this study broaden the overall understanding of the plasma-virus interactions, as well as advocate for a novel non-thermal and chemical-free method of AAV6 disinfection.

## Methods

### Plasma source and virus treatment

**Fig. 6:**
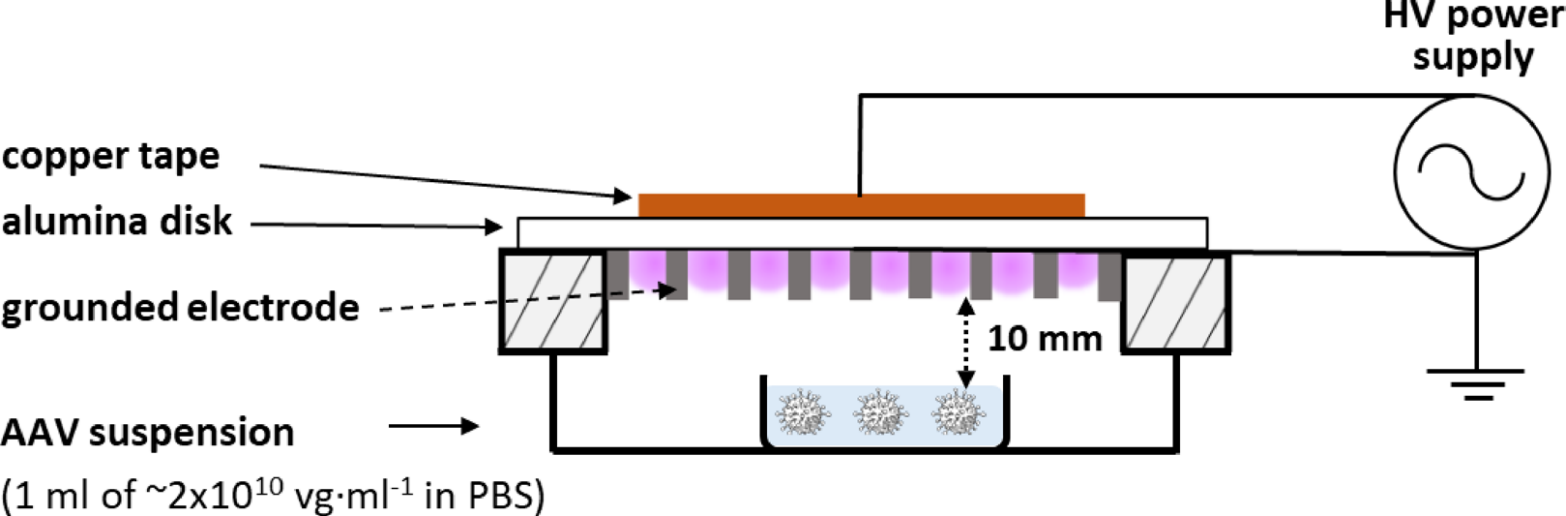
Schematic of the SDBD applied to the AAV6 suspension.

The experimental scheme is shown in Fig. 6. The SDBD consisted of a 0.2 mm thick copper tape high voltage (HV) electrode, a 1 mm thick alumina disk dielectric layer, and the grounded electrode – a 0.5 mm thick stainless steel perforated disk with a diameter of 80 mm and a mesh size of 2 mm. The SDBD was powered by a commercially available power supply customized for this application, which provided an 8.8 kV peak-to-peak voltage waveform at 21 kHz, resulting in an average power of ~ 35 W measured by Lissajous figures. For detailed specifications about the SDBD, the reader is referred to reference^27^.

A vector encoding the green fluorescent protein (GFP) under the control of the cytomegalovirus (AAV6-CMV-GFP) was used in this work. The transgene was packaged into serotype 6 AAV particles by transient transfection of human embryonic kidney (HEK293) cells adapted to culture in suspension (HEKExpress™, ExcellGene SA). Particles were purified from the cell lysate and supernatant using POROS™ CaptureSelect™ AAVX chromatography (Thermo Fisher), concentrated and resuspended in PBS plus 0.001% Pluronic-F68 using centrifugal filter tubes Amicon^®^ Ultra-15 (100 kDa MWCO, Millipore)^35^. The resulting AAV6 vector suspensions were titered by dPCR (QIAcuity, QIAGEN) using a set of primers specific for the intronic β-globin sequence. The concentration of genome-containing particles (vg) in AAV6 preparations were between 2.1×10^12^ vg**·**ml^− 1^ and 2.5×10^12^ vg**·**ml^− 1^.

For plasma treatment, AAV6 was resuspended in PBS to a viral concentration of 2×10^10^ vg**·**ml^− 1^. 1 ml of the viral suspension in a glass Petri dish with d = 30 mm was used for treatment. The gap between the grounded electrode and the liquid surface was kept constant at 10 mm.

### *In vitro* testing of virus infectivity

HEK293 cells were seeded into 24-well tissue culture plate at 2* × *10^5^ cells per well in 500 µl of Serum-free BalanCD^®^ HEK293 Medium (Irvine Scientific) supplemented with 4 mM L-alanyl-L-glutamine (Gibco Glutamax). Cells were transduced 5 h later with control and plasma treated AAV6 samples at a multiplicity of infection (MOI) of 5500 vg**·**cell^− 1^. At 48 h post-infection, cells were stained with DAPI (4′,6-diamidino-2-phenylindole) to exclude false GFP-positive cells, followed by cytometric analysis. The detailed protocol of the infectivity assay can be found in the Supplementary material [S1]. Data were acquired by Attune CytPix Flow Cytometer (Thermo Fisher Scientific) using 488/530-nm (Blue laser) and 405/450-nm (Violet laser) excitation/emission filter for GFP and DAPI, respectively. 10,000 events per sample were acquired. After evaluating the percentage of GFP-positive cells, the numbers of transduction units (TUs) per cell were calculated. For this, a logarithmic relationship (Eq. 9) tied to a Poisson distribution (Eq. 10) was used to model the number of virus particles infecting each cell. Virus inactivation upon plasma treatment was expressed as the fold reduction (*N/N*_*0*_) in virus infectivity, where *N* is the TU of plasma-treated AAV6 and *N*_*0*_ is the TU of untreated AAV6.

9 $$\:TU=\mathrm{ln}(-1/(\frac{GFP}{100}-1\left)\right)$$

where %GFP represents the percentage of the GFP-positive (infected) cells.

10 $$\:P\left(k\right)=\frac{{\mathrm{e}}^{-{\uplambda\:}}\:\times\:\:{\lambda\:}^{k}}{\mathrm{k}!}$$

where *λ* is an average number of TUs, *k* is the number of transgenes per cell, *e* = 2.718, $\:{e}^{-\lambda\:}$ is a fraction of non-infected cells ( $\:\frac{\% GFP}{100}-1$). The limit of detection of the assay was 3 log_10_ TU**·**ml^− 1^.

### Vector genome quantification

**Fig. 7:**
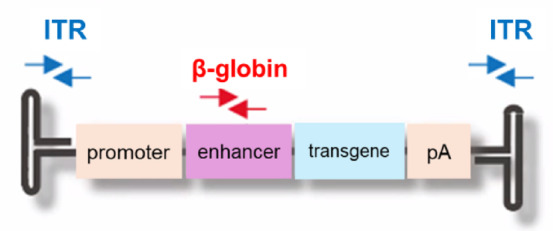
AAV6-CMV-GFP vector: Adeno-Associated Viral (AAV) vector encoding the green fluorescent protein (GFP) under the control of the cytomegalovirus (CMV). ITR: inverted terminal repeat. pA: polyadenylation site.

The impact of plasma treatment on AAV6 particle integrity was measured by dPCR, which allows the absolute quantification of the concentration of genome-containing AAV particles. To ensure viral genome encapsidation, a 15 min DNase I treatment (10 U**·**µl^− 1^, Invitrogen) was applied before multiplexed dPCR, which was performed with TaqMan primers specific for the inverted terminal repeat sequence (ITR, HEX probe; QIAGEN) and the β-globin sequence (FAM probe). 40 cycles of amplification were performed. Vector genome integrity was evaluated by multiplexed dPCR using amplicons located in the single stranded β-globin sequence and in the double-stranded ITR sequences (Fig. 7).

### Transmission electron microscopy (TEM)

For virus visualization, AAV6 particles were resuspended in PBS to a final concentration of 4.7×10^11^ vg**·**ml^− 1^ and a 50 µl sample was treated by plasma for 5, 10, 20 and 60 s. The AAV6 samples were analyzed by the negative staining TEM. The sample was adsorbed on freshly glow-discharged carbon-coated copper grids (400 mesh, EMS, Hatfield, PA, USA), washed with deionized water, and stained with 1% uranyl acetate for 30 s. The grids were observed using the Tecnai F20 electron microscope (Thermo Fisher, Hillsboro, USA), operating at 200 kV. Digital images were collected using a Falcon III camera (Thermo Fisher, Hillsboro, USA) with 4098ˣ4098 pixels, with a defocus range between − 1.5 μm and − 2.5 μm.

### Physicochemical characterization of plasma-activated PBS (PAPBS)

PAPBS was produced by the plasma treatment of 1 ml sterile PBS for 2.5, 5, 7.5, and 10 min. The physicochemical properties of PAPBS were evaluated in the absence of virus by measuring the pH (Unitrode with Pt1000, 6.0258.600 Metrohm) and the temperature (Extech TM26 Temperature Indicator). Concentrations of NO_2_^−^ and NO_3_^−^ in PAPBS were measured by vis-spectrophotometry (Spectroquant^®^ Prove 100, Merck). NO_2_^−^ was measured by the Spectroquant^®^ Nitrite Cell Test (1.00609.0001) with an associated accuracy of ± 2.8 mg·l^− 1^. In acidic solution, NO_2_^−^ reacts with Fe^2+^ ethylenediammonium sulfate to form a yellow to green-brown Fe^2+^ compound. NO_3_^−^ was measured by the Spectroquant^®^ Nitrate Cell Test (1.14563.0001) with an associated accuracy of ± 0.5 mg·l^− 1^. In sulfuric and phosphoric acid solutions, NO_3_^−^ reacts with 2,6-dimethylphenol (DMP) to form 4-nitro-2,6-dimethylphenol, a pink compound. H_2_O_2_ was measured by the Spectroquant^®^ Hydrogen Peroxide Test (1.18789.0001) using the 10 mm quartz cell with an associated accuracy of ± 0.07 mg·l^− 1^. This method is based on the reduction of Cu^2+^ to Cu^+^ by H_2_O_2_ in the presence of phenanthroline derivative, forming an orange complex.

### Statistical analysis

Each experiment was performed independently three times. Values are presented as the mean ± standard deviation. Statistical analysis and visualization of the results were carried out in Microsoft Excel. The statistical significance among groups was evaluated by one-way analysis of variance (ANOVA) and Student’s two-tailed t-test.

## Supplementary Information

Below is the link to the electronic supplementary material.

Supplementary Material 1

## Data Availability

All data supporting the findings of this study are available within the paper and its Supplementary Information. Raw data will be made available from the corresponding author on request.
