# Supplementary material for: Reactive species generated by a cold atmospheric plasma inactivate AAV through oxidative and nitrosative stress
Source: Sci Rep. 2026 May 9;16:21259. doi: 10.1038/s41598-026-52499-3 (PMC13346634; doi:10.1038/s41598-026-52499-3)
Supplement: Supplementary file 1 — Supplementary Material 1 [file 41598_2026_52499_MOESM1_ESM.pdf]

In vitro testing of AAV infectivity

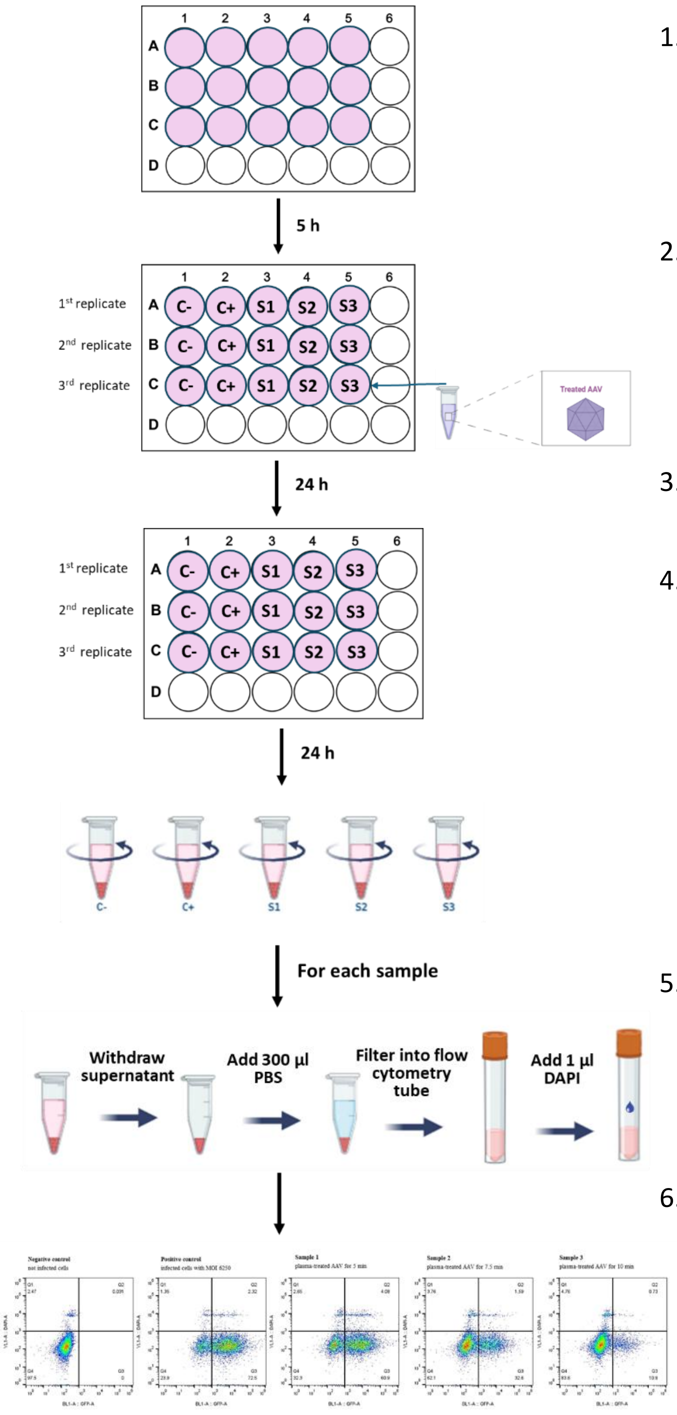

1. Seed HEK293 cells into 24-well tissue culture plates at 200’000 cells/well in 500 µl of medium - Serum-free BalanCD® HEK293 Medium (Irvine Scientific) supplemented with 4 mM L-alanyl-L-glutamine (Gibco Glutamax). Cells are incubated at 37°C with 5% CO_2.

2. When cells adhere (~5 h), decrease volume of the medium in each well to 200 µl and infect cells by adding 50 µl of virus suspension.

   - All samples are performed in triplicate. The final volume per well is 250 µl.

3. After 24 h of infection, add gently 250 µl of medium to each well.

4. At 48 h of infection, cells are prepared for flow cytometry analysis as follows:
   - collect cells from each well and transfer them into Eppendorf tubes;
   - centrifuge cells at 2000 rpm for 2 min, remove supernatant, and resuspend a pellet in 300 µl of PBS;
   - transfer cells to flow cytometry tubes by filtering them through the filter cap;
   - add DAPI (1 µl of a 1 mg/ml stock solution in PBS) to each sample to stain non-viable cells, gently mix.

5. Data acquisition by Attune CytPix Flow Cytometer (Thermo Fisher Scientific) using 488/530-nm (Blue laser) and 405/450-nm (Violet laser) excitation/emission filter for GFP and DAPI, respectively. A minimum of 10.000 events per sample are acquired.

6. Analyze data by FlowJo software.
